# Capivasertib-Induced Diabetic Ketoacidosis in a Non-diabetic Patient With Metastatic Prostate Cancer With Bone Involvement: A Case Report of a Rare but Serious Metabolic Complication

**DOI:** 10.7759/cureus.81513

**Published:** 2025-03-31

**Authors:** Bola Habeb, Otto Valdes, Sandy Khair, Gurmanpreet Sidhu, Matthew Fowler

**Affiliations:** 1 Internal Medicine, University of Florida College of Medicine/Ascension Sacred Heart, Pensacola, USA; 2 Radiation Oncology, Cairo University/National Cancer Institute, Cairo, EGY

**Keywords:** akt inhibitor's adverse effect, capivasertib, chemotherapy-related toxicity, diabetic ketoacidosis (dka), drug-induced dka, medication-induced side effects, metabolic complication, resistance to insulin, targeted cancer therapy

## Abstract

Capivasertib, a protein kinase B (AKT) inhibitor manufactured by AstraZeneca pharmaceutical and used in the treatment of various malignancies, has been implicated in cases of drug-induced diabetic ketoacidosis (DKA). We present a case of capivasertib-induced DKA in a patient with no prior history of diabetes, highlighting the metabolic complications associated with this targeted therapy. The proposed mechanism involves AKT inhibition leading to impaired insulin signaling, reduced glucose uptake, and increased lipolysis, ultimately resulting in ketogenesis. This case underscores the need for vigilant glucose monitoring in patients receiving capivasertib, especially those with predisposing risk factors for insulin resistance or pancreatic dysfunction.

## Introduction

Diabetic ketoacidosis (DKA) is a serious metabolic emergency typically seen in patients with diabetes mellitus; however, emerging reports suggest that certain targeted therapies, including AKT inhibitors such as capivasertib, can induce DKA even in non-diabetic individuals [1]. Capivasertib, an oral selective AKT inhibitor, was approved by the U.S. Food and Drug Administration (FDA) on November 16, 2023, for the treatment of hormone receptor-positive, HER2-negative breast cancer [2]. Additionally, capivasertib has been evaluated in clinical trials for the treatment of metastatic prostate cancer [3]. It works by disrupting the phosphoinositide 3-kinase (PI3K)/protein kinase B (AKT)/mammalian target of rapamycin (mTOR) pathway, which is crucial for regulating glucose metabolism [1,2,4].

Common side effects of capivasertib include diarrhea, nausea, vomiting, mouth sores, fatigue, and alterations in certain blood tests. Skin reactions such as rashes, redness, and blistering may also occur. In addition, more serious side effects like elevated blood sugar levels, kidney problems, and reduced blood cell counts are possible [5]. Hyperglycemia is a well-documented side effect of chemotherapy drugs (e.g., cyclophosphamide and doxorubicin), targeted therapies (e.g., imatinib and nilotinib), and immunotherapies (e.g., pembrolizumab and nivolumab) [6]. In the case of capivasertib, around 16% of patients experience hyperglycemia, although severe, life-threatening hyperglycemia is rare, occurring in only 0.3% of cases, with only a few instances reported in the literature [4].

Patients typically present with symptoms such as polyuria, polydipsia, nausea, vomiting, and altered mental status, often accompanied by severe metabolic acidosis, hyperglycemia, and ketonemia [4]. The diagnostic workup includes serum glucose, arterial blood gas analysis, beta-hydroxybutyrate levels, and a comprehensive metabolic panel to assess for anion gap metabolic acidosis. Management follows standard DKA treatment protocols, including aggressive intravenous (IV) fluid resuscitation, insulin therapy, and electrolyte replacement, while discontinuation or dose adjustment of capivasertib may be required based on severity [7].

This case emphasizes the importance of recognizing acute, severe hyperglycemia as a potential adverse effect of capivasertib and similar cancer therapies. Close blood glucose monitoring at least twice a week is vital to detect any degree of capivasertib-induced hyperglycemia and maintain a high index of suspicion for this as a contributing factor in cases of severe hyperglycemia [4].

## Case presentation

We present a case of a 71-year-old African American male patient with metastatic prostate cancer with bone involvement, atrial fibrillation, and chronic kidney disease stage IIIb who presented to our facility accompanied by his wife for evaluation of altered mental status, worsening generalized weakness, increased thirst sensation, and increased urination for the last week.

Patient information

The patient was diagnosed with metastatic prostate cancer with bone involvement six years ago. He initially received chemo-hormonal therapy with docetaxel and androgen deprivation therapy (ADT). However, disease progression occurred, as indicated by rising prostate-specific antigen (PSA) levels and abnormal findings on a positron emission tomography (PET) scan. Following this, multiple lines of treatment, including enzalutamide (Xtandi), leuprolide (Lupron), and abiraterone acetate (Zytiga), were administered interchangeably. Despite these efforts, further progression was observed, leading to the initiation of cabazitaxel (Jevtana), which was poorly tolerated due to the development of pancytopenia. Genetic testing (Guardant360 (Guardant Health Inc., Redwood City, CA, US)) identified a PTEN (phosphatase and tensin homolog deleted on chromosome 10) mutation, prompting the start of capivasertib (400 mg twice daily, days 1-4, every seven days) six months ago.

Clinical findings

Physical examination revealed a temperature of 37.2°C, blood pressure of 142/78 mmHg, pulse of 89 beats per minute (bpm), 18 breaths per minute, and oxygen saturation of 99% on ambient air. He appeared confused, alert, and oriented only to place and person. The abdominal exam was normal, with soft, lax abdomen, and active bowel sounds. The cardiovascular exam was normal, with a regular rate and rhythm and no audible murmurs. Pulmonary examination demonstrated clear lungs to auscultation with no wheezing, rales, or rhonchi.

Diagnostic assessment

Admission laboratory data are demonstrated in Table 1, compared to laboratory results before initiating capivasertib.

**Table 1:** Laboratory results upon admission, compared to those prior to the initiation of capivasertib pH: potential of hydrogen; pCO_2_: partial pressure of carbon dioxide; pO_2_: partial pressure of oxygen; A1C: glycated hemoglobin *Abnormal lab values

Parameters	Patient’s values on admission	Patient’s values before starting capivasertib	Reference range, adults
Hemoglobin (g/dL)	8.9*	8.4*	12.0–15.5
White cell count (per mm^3^)	14,700*	12,100*	3,500–10,500
Platelet count (per mm^3^)	275,000	60,000*	150,000–450,000
Sodium (mEq/dL)	133	140	135–145
Potassium (mEq/dL)	5.3	4.7	3.5–5.1
Creatinine (mg/dL)	5.34*	1.83*	0.7–1.2
pH	7.22*	7.37	7.31–7.41
pCO_2_ (mmHg)	28.6*	42	35–45
pO_2_ (mmHg)	76	72	60-80
Bicarbonate (mEq/dL)	8*	22	22–29
Anion gap (mmol/L)	25*	8	4-12
Blood glucose (mg/dL)	539*		70-99
A1C	10.9*	5.6	≤6.5
Beta-hydroxybutyrate (mmol/L)	5.68*		0.02-0.27

**Figure 1:**
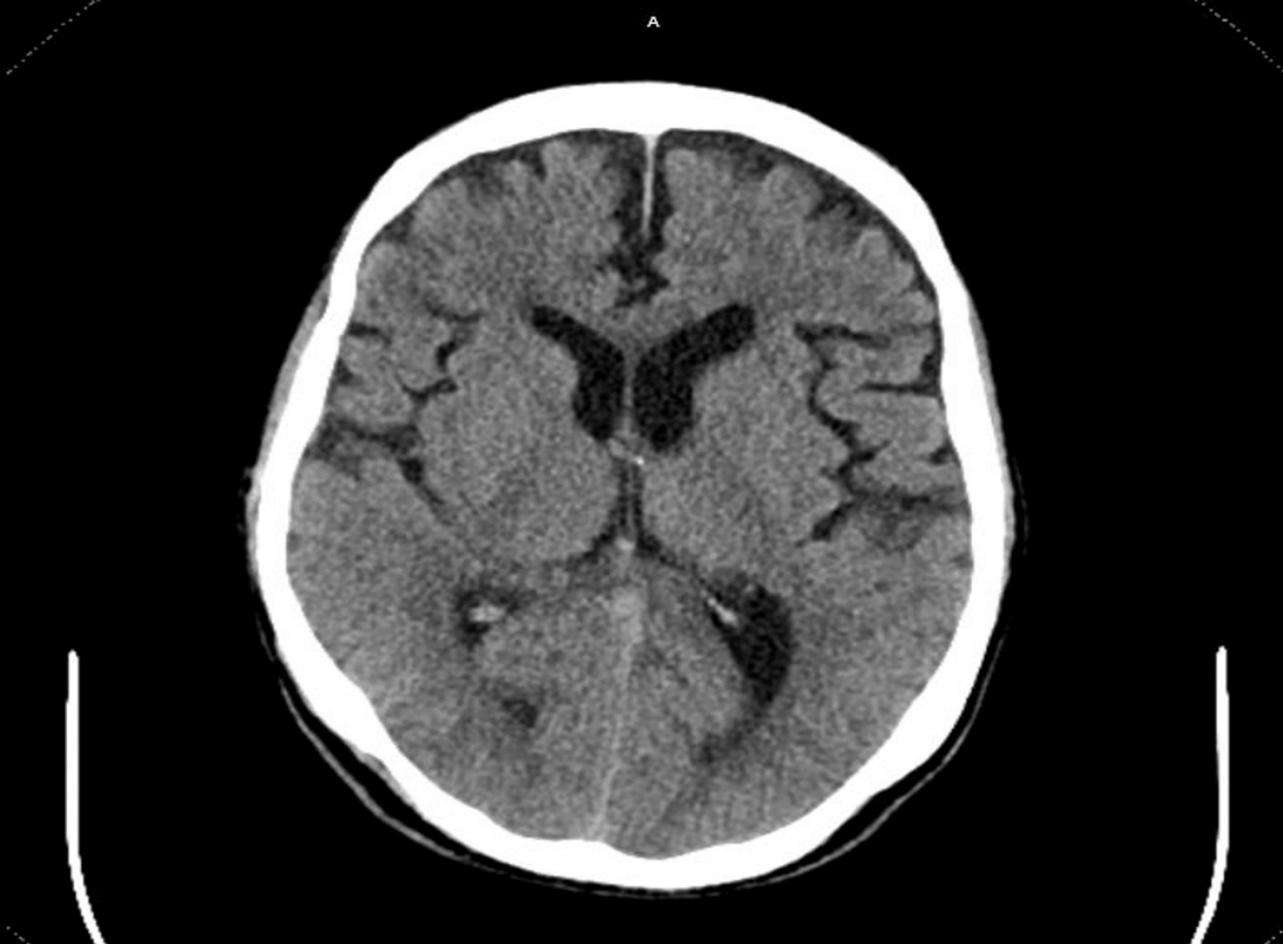
Computed tomography (CT) of the brain without intravenous contrast Axial brain scans reveal no signs of acute intracranial pathology. This includes a normal density of the brain parenchyma, the absence of acute intracranial hemorrhage, and no indications of abnormal masses, growths, mass effect, midline shift, or hydrocephalus.

**Figure 2:**
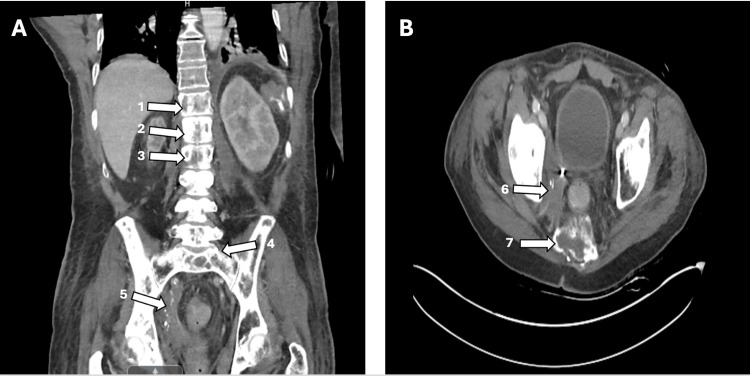
Computed tomography (CT) of the abdomen and pelvis with intravenous contrast (A) Coronal view showing extensive metastatic osseous disease involving the thoracolumbar vertebrae, pelvic bones (arrows 1-4), and right pelvic sidewall soft tissue mass (arrow 5). (B) Axial view showing right pelvic sidewall soft tissue mass (arrow 6) and metastatic osseous disease (arrow 7).

The urinalysis shows 30 mg/dL of protein, >500 mg/dL of glucose, 20 mg/dL of ketones, +3 leukocyte esterase, positive nitrites, >100 HPF white blood cells, and +4 bacteria, which is consistent with proteinuria, glucosuria, ketonuria, and a urinary tract infection. Urine and blood cultures were obtained. The computed tomography (CT) scan of the brain was negative for any acute intracranial abnormality (Figure 1). The CT abdomen and pelvis with IV contrast showed an extensive osseous metastatic disease and a right pelvic sidewall mass with an indwelling right ureteral stent (Figures 2A, 2B).

**Figure 3:**
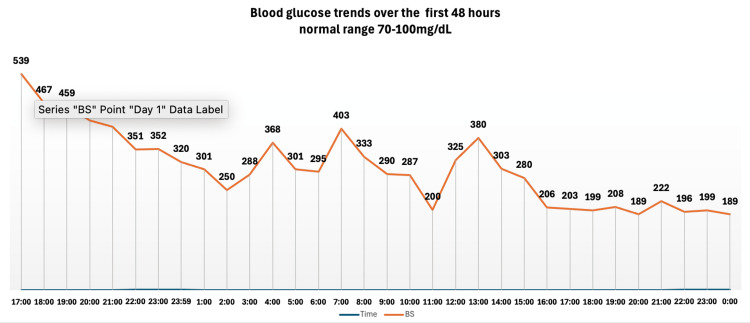
Blood glucose levels during the first 48 hours while receiving an insulin infusion

The elevated anion gap metabolic acidosis, ketosis, and hyperglycemia were consistent with DKA. Considering the normal HbA1c level before initiating capivasertib, the symptom onset after starting capivasertib therapy, known to cause hyperglycemia and insulin resistance, and the exclusion of alternative DKA triggers (e.g., sepsis and pancreatitis), a diagnosis of capivasertib-induced DKA was made. IV fluids and insulin therapy (insulin drip at 0.1 U/kg/hour rate and titrated following the DKA protocol of our institution) were initiated to address hyperglycemia. IV piperacillin/tazobactam was started for empiric broad-spectrum coverage. Urine and blood cultures tested positive for extended-spectrum beta-lactamase (ESBL) *Escherichia coli* (*E. coli*). Consequently, the antibiotic regimen was escalated to IV meropenem with recommendations to continue a two-week course post-discharge. The patient's blood glucose level remained high for the first 48 hours despite continuous insulin infusion via insulin drip (Figure 3).

On day three of admission, the patient's anion gap decreased to eight, with a corresponding improvement in acidosis. He successfully transitioned off the insulin infusion and was initiated on a basal/bolus insulin regimen, with blood glucose levels maintained below 200 mg/dL. Additionally, his renal function returned to baseline, and his level of consciousness showed significant improvement. Repeat blood cultures were negative for microbial growth. The patient was evaluated by a multidisciplinary team, including endocrinology, oncology, and infectious disease specialists, who contributed to managing and stabilizing his condition. He was discharged with recommendations to discontinue capivasertib until further evaluation by an outpatient oncology specialist, continue the insulin basal/bolus regimen, and complete a two-week course of IV meropenem following discharge.

## Discussion

Capivasertib is an FDA-approved oral selective AKT inhibitor to treat hormone receptor-positive, HER2-negative breast cancer [2]. Additionally, it has been evaluated in clinical trials for metastatic prostate cancer, demonstrating its potential as a therapeutic option. The CAPItello-281 Phase III trial tested capivasertib in combination with abiraterone and ADT in patients with PTEN-deficient metastatic hormone-sensitive prostate cancer (mHSPC), enrolling 1,012 patients. The study found that this combination significantly improved radiographic progression-free survival compared to abiraterone and ADT alone [8]. Similarly, the CAPItello-280 Phase III trial assessed capivasertib combined with docetaxel versus placebo and docetaxel in metastatic castration-resistant prostate cancer (mCRPC) patients. Preliminary results suggested that capivasertib, when added to docetaxel, led to improved progression-free survival and showed potential for increasing overall survival [9]. These trials underscore the potential of capivasertib, especially in tumors with PTEN deficiency, as a promising treatment option in metastatic prostate cancer.

Capivasertib specifically targets and inhibits AKT, a key protein within the PI3K/AKT/mTOR pathway, which is crucial for cancer cells' survival and proliferation [3]. AKT is activated by the PI3K enzyme and helps cancer cells grow and evade normal cell death mechanisms. By inhibiting AKT, capivasertib disrupts this signaling pathway, leading to reduced tumor cell growth, as the cancer cells can no longer receive the survival and growth signals they need (Video 1). Additionally, capivasertib induces apoptosis by activating cell death pathways, causing cancer cells to die. Moreover, when combined with other treatments like chemotherapy or hormonal therapies, capivasertib enhances the sensitivity of cancer cells, making them more vulnerable to these treatments [3].

**Video 1:** Capivasertib mechanism of action Source: Reference [10]

By blocking AKT, capivasertib disrupts these processes, not just in cancer cells but also in normal cells, leading to unintended consequences. For example, the inhibition of AKT can affect the regulation of glucose metabolism, leading to elevated blood sugar levels. It can also impact cell turnover in tissues like the skin and gastrointestinal tract, causing symptoms like rashes, mouth sores, diarrhea, and nausea. Additionally, capivasertib may interfere with the production of blood cells in the bone marrow, leading to a decrease in red and white blood cells or platelets, which can result in fatigue, increased risk of infection, or bleeding [5]. Hyperglycemia has been reported in 16% of patients using capivasertib, though severe, life-threatening cases such as DKA or hyperosmolar hyperglycemic state occur in only 0.3% of patients [4]. AKT inhibition is believed to be the primary cause of hyperglycemia associated with capivasertib, as AKT plays a key role in insulin signaling by facilitating tissue glucose uptake and promoting hepatic glycogen storage [4].

In this case, the patient presented with classic signs of hyperglycemia and DKA, including polyuria, polydipsia, and altered mental status. Laboratory results showed elevated blood glucose levels in the 500-600s mg/dL, metabolic acidosis with a pH of 7.22 and bicarbonate level of 8 mEq/L, ketonuria > 20 mg/dL, elevated beta-hydroxybutyrate at 5.68 mmol/L, high anion gap at 25, and an increased A1C level of 10.9%, compared to 5.6% six months prior to starting capivasertib. Given these findings, the patient's DKA is most likely induced by capivasertib. Management involves stopping capivasertib and implementing standard DKA treatment, which includes fluid replacement, insulin therapy, electrolyte management, and correction of acidosis [4].

This report aims to raise awareness among clinicians about hyperglycemia as a potential side effect of capivasertib in both diabetic and non-diabetic patients. The manufacturer advises monitoring fasting blood glucose at least twice a week until it is properly controlled [4].

## Conclusions

Given the expanding use of AKT inhibitors in oncology, clinicians should be aware of the potential for severe hyperglycemia and DKA, even in patients without a history of diabetes. Routine glucose monitoring and early intervention strategies may help mitigate these risks and improve patient outcomes. Further research is needed to better understand the mechanisms underlying capivasertib-induced metabolic derangements and to establish guidelines for risk assessment and management in vulnerable patient populations. This case serves as a reminder that targeted therapies, while effective in cancer treatment, can have significant systemic effects requiring a multidisciplinary approach to care.
